# Hypoxia-Inducible Factor and Its Role in the Management of Anemia in Chronic Kidney Disease

**DOI:** 10.3390/ijms19020389

**Published:** 2018-01-29

**Authors:** Joshua M. Kaplan, Neeraj Sharma, Sean Dikdan

**Affiliations:** Division of Nephrology and Hypertension, Rutgers-New Jersey Medical School, University Hospital, 185 South Orange Avenue, I512, Newark, NJ 07103, USA; neesharma@yahoo.com (N.S.); sean.dikdan@jefferson.edu (S.D.)

**Keywords:** anemia, chronic kidney disease, hypoxia-inducible factor, prolyl-hydroxylase, erythropoiesis-stimulating agent

## Abstract

Hypoxia-inducible factor (HIF) plays a crucial role in the response to hypoxia at the cellular, tissue, and organism level. New agents under development to pharmacologically manipulate HIF may provide new and exciting possibilities in the treatment of anemia of chronic kidney disease (CKD) as well as in multiple other disease states involving ischemia–reperfusion injury. This article provides an overview of recent studies describing current standards of care for patients with anemia in CKD and associated clinical issues, and those supporting the clinical potential for targeting HIF stabilization with HIF prolyl-hydroxylase inhibitors (HIF-PHI) in these patients. Additionally, articles reporting the clinical potential for HIF-PHIs in ‘other’ putative therapeutic areas, the tissue and intracellular distribution of HIF- and prolyl-hydroxylase domain (PHD) isoforms, and HIF isoforms targeted by the different PHDs, were identified. There is increasing uncertainty regarding the optimal treatment for anemia of CKD with poorer outcomes associated with treatment to higher hemoglobin targets, and the increasing use of iron and consequent risk of iron imbalance. Attainment and maintenance of more physiologic erythropoietin levels associated with HIF stabilization may improve the management of patients resistant to treatment with erythropoiesis-stimulating agents and improve outcomes at higher hemoglobin targets.

## 1. Introduction

Anemia is a common feature in patients with chronic kidney disease (CKD). Although optimal treatment of this condition remains controversial, for many years, erythropoiesis-stimulating agents (ESAs) have been utilized and perhaps considered the standard of care. Partial correction of severe anemia with these agents has been shown to provide improvement in cardiovascular parameters and quality of life (QoL) [1,2,3].

Current guidelines support only partial correction of anemia in patients on dialysis; full hemoglobin normalization by ESAs did not benefit CKD patients in multiple studies [1]. Many patients with anemia of CKD receive intravenous (IV) iron supplementation, which also presents safety concerns [4]. 

One class of agents under development for the management of anemia in CKD acts by stabilizing hypoxia-inducible factor (HIF) through inhibition of the prolyl hydroxylase family of enzymes. New agents under development to pharmacologically manipulate HIF have been investigated.

Eukaryotic organisms must live in an environment containing oxygen to facilitate the efficient creation of energy necessary for life processes. However, due to the capacity for cytotoxic damage, the oxygen supply must be tightly regulated. HIF, a transcription factor that plays an important role in the cellular response to systemic oxygen levels, provides a primary means by which systemic oxygen delivery to mammalian cells can be regulated through hemoglobin content. Moreover, on a basic level, HIF also mediates a response to oxygen deficiency at cell, tissue, and systemic levels. This review discusses these responses to hypoxia, the role of the HIF pathway, and ways in which the HIF pathway can be manipulated in the clinical setting.

## 2. Hypoxia-Inducible Factor

Key processes in CKD underlying the development of anemia are erythropoietin (EPO) deficiency and functional iron deficiency (FID), typically managed with use of iron, blood transfusion, or administration of an exogenous ESA. Novel clinical agents targeting the HIF pathway stimulate endogenous EPO production while simultaneously coordinating iron bioavailability. These agents exhibit potential for a more physiologic erythropoietic response, with red blood cell (RBC) production stimulated by physiologic levels of EPO rather than high intermittent blood levels associated with exogenous ESA treatments [5]. 

Extensive efforts towards understanding the regulation of erythropoiesis and EPO mRNA resulted in the identification of HIF in 1992, which was subsequently purified by Semenza and Wang in 1995 [6,7]. Characterization of HIF as a heterodimeric hypoxia-inducible DNA-binding protein comprising an alpha and beta subunit, that activated EPO gene transcription, soon led to the suggested existence of a common hypoxia signal transduction pathway and a central role for HIF in the transcriptional regulation of hypoxia-responsive genes [7]. In addition to activating EPO gene transcription, HIF has since been shown to activate a number of genes involved in iron uptake and transport, resulting in coordinated erythropoiesis that involves stimulation of endogenous EPO production, increased iron availability for proerythrocytes, and promotion of maturation of erythrocytes replete with hemoglobin [8].

The alpha and beta subunits of HIF bind together in the cell nucleus to form a functional dimer which, in the presence of various cofactors, binds to DNA sequences or hypoxia response elements (HREs) to induce expression of target genes. Notably, the beta subunit is generally present in excess, while the alpha subunit represents the limiting factor in creation of the functional dimer. 

HIF subunits exists in three α isoforms, HIF-1α, HIF-2α, and HIF-3α, and as an HIF-β isoform also known as aryl hydrocarbon receptor nuclear translocator (ARNT). After dimerization in the nucleus, HIF directly activates renal and hepatic erythropoietic genes, resulting in coordinated erythropoiesis including stimulation of endogenous EPO production, upregulation of transferrin receptor expression, increased iron uptake by proerythrocytes, and promotion of maturation of erythrocytes replete with hemoglobin. The tissue expression of HIF-1α mRNA is generally ubiquitous [9], while mRNA expression of HIF-2α is mainly in the brain, heart, lung, kidney (interstitial and glomerular renal cells), liver, pancreas, and intestine [9,10,11]. Specifically, HIF-2α is required for renal and hepatic expression of EPO [12], coincidentally, the main organs of EPO production. While the relevance of tissue and intracellular distribution of the HIF isoforms remains to be established, it is reasonable to suppose that not all tissues and cells will be equally exposed to a pharmacologically administered therapy; furthermore, drug kinetics, distribution, and excretion may also differ between tissues. The tissue expression of HIF-3α remains unclear, although hypoxia-induced increased expression has been reported in the heart, lung, and kidney [13].

HIF-α subunits are constantly targeted for hydroxylation at two proline residues by a family of prolyl-hydroxylase enzymes, designated as prolyl-hydroxylase domain PHD1, PHD2, and PHD3 [14,15]. These hydroxylase enzymes have a distinct pattern of intracellular localization in human tissues with PHD1 and PHD2 localized mainly in the cytoplasm, and PHD3 in both the cytoplasm and nucleus [16]. PHD2 has also been shown to shuttle between the cytosol and the nucleus [17]. The relevance of the intracellular distribution of the PHD isoforms remains to be established, although reporter assay studies (albeit using tagged non-native proteins) indicate the intracellular distribution of PHD1 does not influence HIF-1α activity, while regulation of the intracellular distribution of PHD2 between the cytoplasm and the nucleus is an effective pathway for the control of the hypoxic response [17].

Hydroxylation at one or both proline sites results in the binding of HIF-α by the von Hippel-Lindau tumor suppressor protein (pVHL), part of a complex which possesses ubiquitin ligase E3 activity, resulting in rapid ubiquitination and proteosomal degradation [18]. Additionally, HIF-α subunits may be hydroxylated at a C-terminal asparagine residue by factor inhibiting HIF (FIH) [19], which regulates HIF-α recruitment of transcriptional coactivators, and transcriptional activation of certain HIF target genes [20,21,22]. Studies of the extent to which HIF target gene expression is induced by PHD or FIH inhibition showed substantial differences in the role of prolyl and asparaginyl hydroxylation in regulating hypoxia-responsive genes, indicating that FIH-regulated coactivators were not required for the therapeutic regulation of some HIF target genes; however, simultaneous inhibition of PHDs and FIH are likely to be required for other therapeutic applications [22,23].

### 2.1. Regulation of HIF

In normoxia, persistently high mRNA levels of all HIF subunits were detectable in a variety of tissues in rats, including the cerebral cortex, hippocampus, lung, and heart, mirroring generally ubiquitous de novo synthesis of HIF proteins under these conditions [9,10,11]. Under normoxic conditions, HIF-1α in particular is rapidly degraded and cannot be detected with immunostaining. However, under hypoxic conditions, HIF-1α remains stable, detectable by immunostaining, and active, increasing gene transcription by binding to HREs along with HIF-1β and various cofactors. Such sensitivity to hypoxia is mediated through the functional dependence of PHD and FIH enzymes on oxygen. Both enzymes require oxygen to effectively hydroxylate HIF-α but are slow to bind and associate with their respective substrates. As the Km (oxygen) of these enzymes is higher than the usual tissue concentration of oxygen, any small decrement in tissue oxygen levels results in decreased function of the PHD enzyme family and, to a lesser extent, FIH, due to a lower Km (oxygen), resulting in increased stability and function of HIF [24]. In addition to primary regulation by oxygen, the activities of PHD and FIH are also dependent on the presence of cofactors ferrous iron and ascorbate, substrate 2-oxoglutarate (aa-ketoglutarate, or 2OG), and inhibition by other citric acid cycle metabolites [25,26,27]. Collectively, these observations suggest HIF activity is dependent on oxygen concentration, accessible iron, and glucose metabolism.

### 2.2. Genetic Determinants of HIF Activity

The effects of HIF activity have been demonstrated through a variety of models (effects of altitude, effects of human mutations, and genetic models in animals). HIF-2α is the most important isoform in the response to anemia, supported by the recent observation that a mutation in the HIF-2α gene, which compromises binding of HIF-2α to PHD2 and VHL, is associated with erythrocytosis (Table 1) [26]. HIF-1α is more important in the response to local ischemia, with effects on angiogenesis and anaerobic metabolism.

In vitro studies demonstrate there is significant diversity in the impact of hypoxia on cell and tissue function and gene expression, relating to a complex interaction of multiple isoforms of HIF-α and PHD. The expression of a variety of genes is modulated by HIF, including those involved in anaerobic metabolism or associated with angiogenesis, those related to RBC production, including EPO and iron-handling proteins, and a variety of other genes. While HIF-2α appears to be the key factor in mediating the response to anemia with an impact on EPO synthesis and iron handling [49,50,52,53,54,55,56,57], the DNA target sequences for HIF-1α and HIF-2α are similar, thus, in vivo there appears to be significant differentiation regarding the downstream effects of the two isoforms [58,59]. In general, HIF-1α appears to be the primary factor in mediating the response to local tissue ischemia and hypoxia, increasing angiogenic factors, blood supply, and the ability to carry out anaerobic glycolysis [60,61,62,63,64,65,66,67,68].

**Figure 1:**
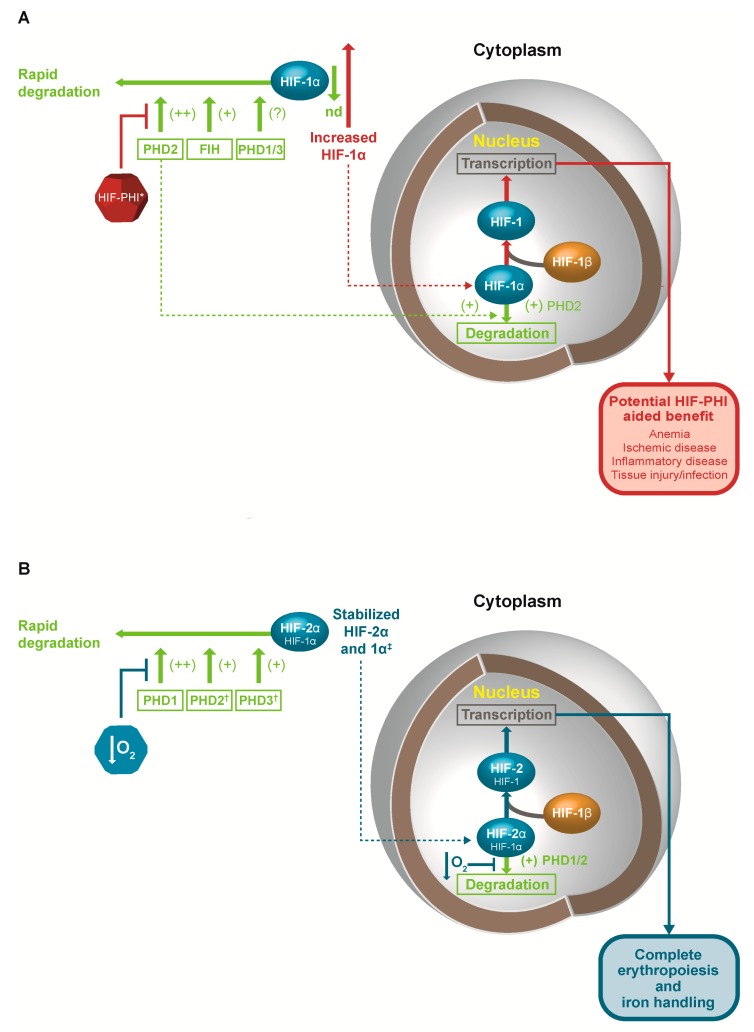
Primary hypoxia-inducible factor (HIF) intracellular distribution and key points of action in (**A**) Normoxia and (**B**) Hypoxia. Green arrows and text represent pathways of degradation of HIF. Red arrows and text represent effect of HIF-PHI, while blue arrows and text represent effect of hypoxia. T-bar represents inhibition of a pathway. Expression: HIF-1α: ubiquitous tissue expression; HIF-2α: brain, heart, lung, kidney, liver, pancreas, and intestine; HIF-3α: heart, lung, and kidney. Specificity: PHD2 and FIH, HIF-1α; PHD1 and PHD3, HIF-2α. * Effect of HIF-PHI on FIH unclear. Dotted lines represent translocation to nucleus. ^†^ PHD2 and PHD3 upregulated by hypoxia as part of a counter-regulatory mechanism. ^‡^ In certain situations, HIF-1α controls the early response to hypoxia. FIH: factor inhibiting HIF; HIF-PHI: hypoxia inducible factor-prolyl hydroxylase inhibitor; nd: not detectable; PHD: prolyl-hydroxylase domain.

The regulation of HIF activity by different PHD isoforms is also based on a complex and overlapping organization (Figure 1). PHD2 is the main regulator of HIF and erythropoiesis, with PHD1 and PHD3 contributing in certain settings. In general, PHD1 expression is constitutive but not induced by hypoxia, while the expression both of PHD2 and PHD3 is induced by hypoxia, having HREs recognized by both HIF-1 and HIF-2 [69]. Moreover, each of the three PHD isoforms has a distinct tissue expression pattern [70], while differences have been observed in the affinity of the different PHD isoforms for the HIFα isoforms. PHD2 shows a marked preference for HIF-1α, and PHD1 and PHD3 show a preference for HIF-2α [69]. Genetic studies have shown that loss of any two of the three genes for PHD isoforms in the liver leads to increased EPO expression and polycythemia, which can be blocked by loss of the HIF-2α gene, but is not blocked by the loss of the HIF-1α gene [71].

In contrast to the three PHD isoforms, the relationship of FIH to RBC production is uncertain. A null mutation in the FIH gene, generated in mice, failed to indicate any role for FIH in classical aspects of HIF function, e.g., erythropoiesis, angiogenesis, or development in the absence of PHD inhibition [46]. Specifically, FIH hydroxylates HIF-α at an asparagine residue, thus altering the affinity of HIF for the p300/CREB-binding protein coactivator complex and blocking activation of affected HREs [18,19,72]. FIH also hydroxylates a variety of other proteins including tankyrase-1, a poly-ADP-ribosyl transferase involved in the cellular regulation of telomere length, apoptosis [73] and proteins in the Notch pathway [74,75,76], which facilitate interaction between the hypoxia-signaling pathway and other related pathways.

Studies in animal models have further elucidated a multifarious impact of the different PHD isoforms on cellular and tissue function (Table 1). Phenotypes of knockout and knockdown mice suggest that PHD1 is involved with the local response to ischemia, while PHD2 is involved with angiogenesis. PHD3 knockout mice have alterations in sympathetic nervous system function, and FIH knockout animals exhibit a metabolic phenotype characterized by reduced body weight, elevated metabolic rate, improved glucose and lipid homeostasis, and resistance to high fat diet-induced weight gain and hepatic steatosis. 

Animal models suggest that HIF-1α impacts on local effects, such as increasing anaerobic metabolic capacity, while HIF-2α relates to global responses to hypoxia, especially erythropoiesis. The clinical impact of mutations in humans provides further support to these characterizations. For example, patients with Chuvash Polycythemia phenotypes (mutations affecting VHL) demonstrated a lack of angiogenic or metabolic phenotypes, with intact hypoxic regulation and no predisposition to tumorigenesis [48,49,77,78].

### 2.3. HIF and Iron Handling

**Figure 2:**
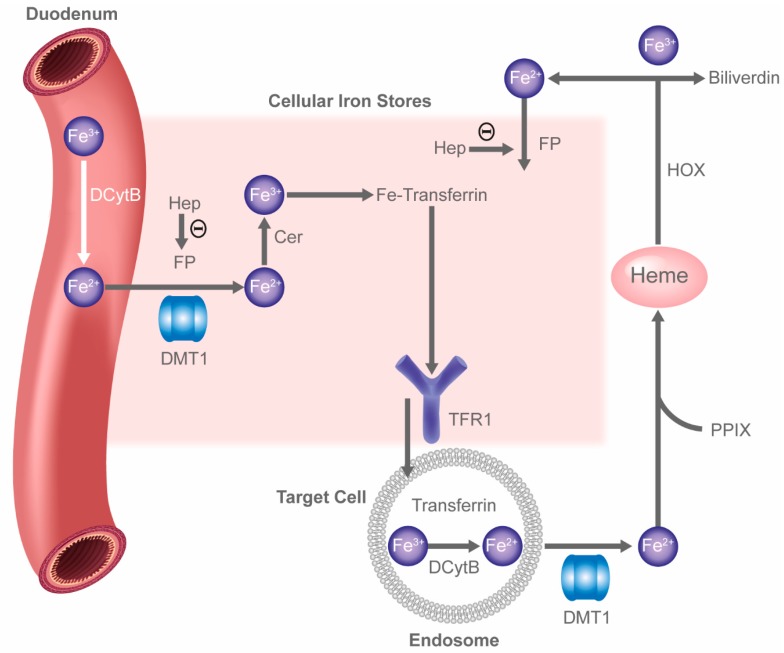
Iron handling. Cer: ceruloplasmin; DCytB: duodenal cytochrome B; DMT1: divalent metal transporter 1; Fe: iron; Hep: hepcidin; HOX: heme oxygenase; FP: ferroportin; PPIX: protoporphyrin IX; TFR: transferrin receptor. Θ represents a negative effect. Reprinted by permission of the publisher Taylor & Francis Ltd. (http:/www.tandfonline.com), from Muchnik & Kaplan [79].

A major class of genes moderated by HIF includes those genes involved in iron handling and metabolism. Iron is critical to multiple cell processes, including erythropoiesis. While most iron requirements are satisfied by recycling of iron in senescent RBCs, iron losses must be compensated for by absorption of dietary iron. Dietary ferric iron must first be reduced to ferrous iron by duodenal cytochrome B (DCytB); ferrous iron is then transported into enterocytes lining the gut via divalent metal transporter 1 (DMT1) (Figure 2). The transport of ferrous iron out of enterocytes into the bloodstream occurs via the iron transporter or ferroportin, which has not been reported to be HIF regulated. Once in the bloodstream, ferrous iron is oxidized to ferric iron by ceruloplasmin and bound for transport by transferrin. Transferrin binds to cellular membrane transferrin receptor 1 (Tfr1) and the iron is internalized by endocytosis. Ferric iron is reduced to its ferrous state for transport out of the endosome by DMT1 and is either stored or released via DMT1 to support cellular functions, including heme formation. This utilization of iron is opposed by the hepatic peptide hormone hepcidin, which reduces dietary iron absorption and blocks release of stored iron both from macrophages and the liver by inhibiting ferroportin, thus reducing the availability of stored iron and potentially promoting anemia. This situation may be compounded in states of infection and systemic inflammation when hepcidin levels are increased by cytokines such as interleukin-6, tumor necrosis factor-α (TNF-α), and interleukin-1. Here, iron per se can be potentially cytotoxic, both as an oxidative moiety or as a cofactor promoting growth of infectious organisms. 

HIF, however, modulates iron handling by upregulating transferrin, Tfr1, and ceruloplasmin [80,81,82,83], allowing greater transport of iron to tissues, and increasing intestinal absorption of iron via upregulation of DCytB and DMT1, and downregulating hepcidin [8]. It has recently been shown that HIF-mediated regulation of hepcidin is dependent on EPO-stimulated erythropoiesis, very likely mediated via erythroferrone secretion from the bone marrow [12,84]. While this is likely the main mechanism by which global hypoxia or HIF stabilization mediates decreases in hepcidin levels, direct effects via HIF-mediated induction of furin or matriptase-2 may also contribute to overall hepcidin suppression by HIF [85,86,87]. Such effects occur primarily via HIF-2α, with HIF-1α having a similar but relatively smaller role [88]. HIF thus increases iron absorption, accessibility to iron stores, and iron transport, while concomitantly opposing any negative impact of iron, either on inflammatory status or infection.

## 3. Current Standard of Care for Anemia in Chronic Kidney Disease (CKD)

Erythropoiesis-stimulating agents and adjuvant IV iron supplementation represent the current standard of care in patients with anemia in CKD [89]. Prior to the introduction of ESAs in 1989, hemodialysis patients required repeated red cell transfusions, which are associated with a number of risks, including blood-borne infections, allosensitization, and infusion-reactions [89]. Subsequently, the introduction of ESAs dramatically changed the landscape of anemia management in renal disease, allowing patients to maintain higher hemoglobin, with decreased cardiovascular events and improved QoL, with fewer transfusions. The introduction of IV iron was the final step to the current standard of care, as achieving maximum effect from use of an ESA requires adequate iron stores, since iron deficiency (either absolute or functional) is the most common cause of ESA hypo-responsiveness. Consequently, iron supplementation remains an integral aspect in the treatment of anemia of CKD.

Iron plays an essential role in many biological pathways, such as oxygen transport, the oxidation and reduction of enzymes, DNA synthesis, and cellular respiration. However, when in excess, iron can prove toxic due to the generation of free radicals. Thus, systemic iron levels are tightly regulated as described earlier (Section 2.3: HIF and iron handling). CKD patients are also at increased risk from iron loss with, for example, hemodialysis patients losing around 1–3 g of iron per year, primarily due to iron loss during dialysis, frequent phlebotomy, surgical procedures, and gastrointestinal losses; an average 70 kg man has about 3.5 g of total body iron [90,91,92]. Consequently, CKD patients are at increased risk of developing absolute iron deficiency along with a functional deficiency mainly due to the chronic systemic inflammatory status typical of CKD patients. Functional iron deficiency is characterized by an inability to sufficiently mobilize iron from storage sites despite adequate iron stores, along with low transferrin saturation and normal to high serum ferritin levels. Recent data have shown elevated hepcidin levels to be a major contributor to the disordered iron homeostasis evident in anemia of CKD [91]. Hepcidin, a major regulator of iron homeostasis, is increased in the presence of inflammation and impairs dietary iron absorption and iron mobilization from storage. 

Accordingly, many patients with CKD, both ESA- or non-ESA-treated, receive IV iron supplementation. However, concerns exist regarding iron toxicity and potential adverse impacts of IV iron on stimulating bacterial growth, increased risk of infection, and direct cellular toxicity [4]. Iron supplementation has some significant drawbacks. Iron holds the potential to generate reactive oxygen species (ROS), such as hydroxyl radicals formed when iron is oxidized from Fe^2+^ to Fe^3+^. Systemically, however, both circulating and cellular iron is protein-bound, or complexed into hemoglobin or myoglobin, which serves to minimize its catalytic activity [4]. Intravenous iron use thus bypasses the normal physiologic safeguards against potential ROS generation. 

The use of IV iron also directly induces oxidative stress and causes cellular injury [93]. Patients with CKD already demonstrate general stress in the form of uremia, acidosis, dialysis, and infection [94,95,96], which may be compounded by iron supplementation used to treat their CKD-related anemia. In patients on dialysis, markers of increased oxidative stress are found to be increased in patients with higher serum ferritin levels [97,98,99]. This is cause for concern given the significant increases both in serum ferritin and iron dosages of dialysis patients recorded in recent years. In one study, the United States Dialysis Outcomes and Practice Patterns Study (DOPPS), 91 facilities measured a significant increase in mean serum ferritin level from 601 ng/mL in 2009 to 887 ng/mL in 2012 [100]. The added systemic stress associated with iron therapy may be causally related to the increased risk of infection, cardiovascular events, worsening diabetes, and cognitive problems that affect patients undergoing dialysis. 

Intravenous iron has also been linked to cardiovascular disease, the major cause of mortality in end stage renal disease (ESRD) patients, with increased serum ferritin reproducibly associated with higher cardiovascular events and overall mortality [101,102,103]. Mirroring this, a large observational trial in hemodialysis patients demonstrated a correlation between increased IV iron dosage/month and increases in mortality rates [103]. Additionally, both human and animal studies have implicated iron therapy further in the hastening of atherosclerosis, suggesting a potential major role in pathogenesis [98,104,105]. Data relating to the risk of infection associated with iron therapy are generally inconsistent, although several large studies have showed a significant correlation, particularly when patients are followed up long term [106]. One study of more than 1000 hemodialysis patients in Japan followed up for over 2 years showed that a modestly elevated serum ferritin of >100 ng/mL was associated with a 76% increase in chance of infection [102]. It is unknown, however, whether the discriminant value for ferritin at which such associations with increased infection are seen is affected by ethnic group or not. Several in vitro studies have shown a negative impact of iron therapy on T-lymphocytes, with induced apoptosis and disruption of the phagocytic potency of polymorphonuclear leukocytes [107,108]. Iron therapy may also worsen the metabolic status of diabetic patients by increasing both oxidative stress on pancreatic β-cells [109] and insulin resistance, and potentially CVD risk, and has been linked to impaired muscle strength and, in case reports, to secondary hemochromatosis [110,111]. Collectively, these observations suggest iron overload remains a growing concern; here radiology, notably quantitative hepatic magnetic resonance imaging, is proving increasingly useful in the early diagnosis of iron overload for dialysis patients [112].

As with IV iron supplementation, ESA use is itself associated with some notable limitations. For example, there is evidence that treatment with ESAs contributes to cardiovascular morbidity, stroke, thrombotic events, and mortality independently of iron use, and worsens hypertension in patients with CKD [3]; complications that resulted in revised US Food and Drug Administration safety guidance for ESAs in 2007. There is a significant rate of hypo-responsiveness to ESAs, resulting in the need for administration of high doses of ESAs. High doses of ESAs, in turn, have been associated with elevated inflammatory biomarkers and increased levels of circulating endogenous soluble EPO receptors (sEpoR), an inhibitor of erythropoiesis [64]. Although rare, there have also been reports of pure red cell aplasia secondary to treatment with an ESA [113]. 

### 3.1. Pitfalls of Current Standards of Care

Anemia is a common feature in patients with CKD, especially when estimated glomerular filtration rate falls below 60 mL/min. The global magnitude of anemia in patients with CKD is considerable; in the US alone, the prevalence is ~15%, representing an estimated 4.8 million people [114]. As renal failure progresses, the frequency of anemia of CKD increases, occurring in up to 95% of patients with ESRD. Anemia of CKD is mainly normocytic and normochromic, and is primarily due to decreased renal EPO production, reduced RBC survival, and decreased responsiveness to EPO [5]. The optimal treatment for anemia of CKD remains surprisingly unclear. It is, however, evident that anemia conveys burden both to the patient and healthcare providers in terms of increased risk of (cardiovascular) morbidity and mortality, the development of left ventricular hypertrophy, and decreased QoL, including the need for transfusion and hospitalization [115,116,117,118,119]. 

### 3.2. Treatment to Hemoglobin Targets

Several clinical studies and meta-analyses have evaluated the benefits or otherwise of normalizing hemoglobin levels in individuals with anemia associated with CKD. Some studies show benefits (mainly cardiovascular and QoL-related) in treating to physiological hemoglobin targets [120,121,122]. Partial treatment of severe anemia of ESRD with ESAs, raising hemoglobin from <10 to ≥10 g/dL, has been shown to provide significant improvement from baseline in these parameters [123,124,125]. It follows intuitively that if anemia increases morbidity and mortality, and partial correction of hemoglobin levels reverses this trend, then complete correction of anemia should improve outcomes even further. However, compared with partial anemia correction, there is much evidence that hemoglobin normalization has no benefit, or can even be harmful in CKD patients [1]. Hemoglobin normalization has been shown to be associated with increased risk of stroke, hypertension, cardiovascular morbidity, and mortality [2,3]. 

The NORMAL-Hematocrit trial randomized hemodialysis patients to a target haematocrit of 42% versus 30%, and found that full correction of hematocrit to 42% was associated with greater risk of mortality and first myocardial infarction [126]. Subsequent re-examination of this question with CREATE, CHOIR, and TREAT again showed no improvement in outcomes, with an increased cardiovascular risk [127,128,129]. A considered post-hoc analysis of the TREAT study data, aimed at identifying the factor(s) associated with increased risk, showed treatment to a higher hemoglobin target to be associated with greater use of IV iron therapy and a higher ESA dose [130]. Based on this, a putative mechanism by which increased doses of IV iron might result in worsened outcomes can be deduced (discussed below) but a causal link could not be established. Interestingly, a connection was found in the Normal Hematocrit Cardiac Trial between outcomes and the dose of ESA where, irrespective of randomization, higher doses of ESA were associated with greater risk of adverse outcomes. The connection between higher doses of ESAs and increased risk of mortality could be because ESA resistance leads to higher doses of ESAs, and increased ESA resistance may correlate with increased risk of mortality. This hypothesis would not explain, however, why patients in the higher hemoglobin target arm did worse than in the lower target arm, given that ESA resistance should be equal between the two arms through randomization. It can be suggested, therefore, that increased doses of ESAs could lead directly to increased mortality. Possible theories are that overstimulation of less specific, lower affinity, widely-distributed ESA receptors [131], or an increase in blood pressure associated with higher doses of ESA, may represent the underlying mechanism for this adverse effect. While the reason for worse outcomes with normalization of hemoglobin remains unclear, the effect has been demonstrated in multiple high-quality studies. Accordingly, current guidelines support partial treatment of anemia in patients on dialysis, with a target hemoglobin above 10.0 g/dL (ideally 11.0–12.0 g/dL), whereas management goals in patients not on dialysis focus on reducing the need for blood transfusion and the management of symptoms of anemia [1].

### 3.3. Hypoxia-Inducible Factor Prolyl-Hydroxylase Inhibitors

Based on exploration of the HIF pathway to date, it would be expected that interference with the function or activity of PHD isoforms would have benefits in anemia in CKD, and possibly other clinical conditions. One emerging and promising clinical strategy is to inhibit the function or activity of PHD isoforms and stabilize HIF using a class of drugs called ‘Hypoxia Inducible Factor Prolyl-Hydroxylase Inhibitors’, or HIF-PHIs. The treatment of anemia in CKD is the primary therapeutic target of HIF-PHIs currently under investigation. The primary focus in terms of adverse effects of HIF-PHIs relates to theoretical concerns about angiogenic effects, which could lead to proliferative retinopathy and certain cancers. There is a connection between HIF and TGF-β, as well as suggestions that HIF activation can lead to renal fibrosis [111,112,113]. However, there are also potential pathophysiologic mechanisms for HIF to reduce renal fibrosis and progression of CKD [113]. The degree of activation of HIF may also differentiate between positive and negative effects of HIF activation. Finally, the primary therapeutic target for treatment of anemia is HIF2α, while many of these other interactions are with HIF1α. With these areas of uncertainty, monitoring the safety data in clinical trials is of paramount importance. As yet, clinical trials have not shown an increased risk of proliferative retinopathy, cancer development or progression, or progressive renal disease. In addition, there is interesting evidence relating to the safety and efficacy of increased HIF activity from studies of populations at high altitude. Long-term observational data showed a decrease in overall cancer-related mortality at high altitudes, with a possible increase in mortality only in prostate cancer. Dialysis patients living at high altitude have higher hematocrits with lower ESA doses and lower relative mortality rates [132,133]. While these studies do not directly study the effects of HIF-PHIs, the results are reassuring for the safety of activating the HIF pathway chronically.

A number of promising compounds are under active investigation; in particular, roxadustat (FG-4592, FibroGen, San Francisco, CA, USA/Astellas, Northbrook, IL, USA/AstraZeneca, Wilmington, DE, USA), daprodustat (GSK1278863, GlaxoSmithKline, Philadelphia, PA, USA), molidustat (BAY85-3934, Bayer, Leverkusen, Germany), and vadadustat (AKB-6548, Akebia Therapeutics Inc., Cambridge, MA, USA) have been shown in phase 2 trials to increase hemoglobin levels in incident dialysis patients [134,135] and non-dialysis CKD patients [136,137,138,139]. This aside, the clinical role of these molecules now needs to be determined in the context of available and effective treatments for anemia of CKD. To establish a potential clinical role for this class of agent, the profiles of established therapeutic approaches in the treatment of anemia of CKD are first considered.

“New agents under development to pharmacologically manipulate HIF present exciting new possibilities in the treatment of anemia of chronic kidney disease.”

### 3.4. Clinical Impact of HIF-PHIs in Anemia of CKD

The chief concerns surrounding the current therapeutic approach to treatment of anemia of CKD are the increased risk of cardiovascular morbidity and mortality when treating to a higher (physiological) hemoglobin target with ESAs, the risk of increased morbidity and mortality with aggressive use of iron repletion, and the management of patients with ESA hyporesponsiveness/resistance or who develop anti-EPO antibodies. It is hypothesized that these concerns may be addressed by the clinical use of HIF-PHIs. A primary reason for use of aggressive iron repletion and consequently elevated ferritin levels is the need to overcome functional iron deficiency despite total body iron excess. As alluded to earlier, such deficiency is due to sequestration of bodily iron stores by hepcidin, which is in turn increased by the pro-inflammatory status of CKD patients [140]. The action of HIF has been shown to drive a coordinated regulatory response resulting in increased iron uptake and decreased hepcidin levels, leading to more effective internal iron metabolism [85,86,87]. Clinical trials of HIF-PHIs have shown decreased levels of both hepcidin and ferritin, the latter reflecting utilization of stored iron, and increased total iron binding capacity, which facilitates transfer of iron to the bone marrow [134,135,137,138,141]. To date, there have been no studies performed on HIF-PHIs specifically in patients with ESA resistance. As the most significant causes of ESA resistance include increased inflammatory status and functional iron deficiency, feasibly, HIF-PHIs may represent an appropriate treatment strategy in these patients, improving functional iron status without the need for excess iron administration, while avoiding the need for high doses of ESAs and their associated cost, and the potential for increased morbidity risk. Similarly, if the hypothesis is borne out that increased cardiovascular morbidity and mortality in normalization of hemoglobin in anemia of CKD results from the use of high doses of ESAs, it is biologically plausible that HIF-PHIs allow the benefits of increased hemoglobin without the risks associated with high-dose ESAs, by normalizing hemoglobin but with much lower peak serum EPO levels compared with high-dose ESA therapy.

“Based on mode of action and available evidence, treatment with HIF-PHIs may allay concerns evident with current standards of care.”

## 4. Other Potential Therapeutic Roles for HIF-PHIs

Although the clinical development of HIF-PHIs is well underway and their role in the treatment of anemia of CKD is being firmly established, recent reports have described a variety of putative pleiotropic beneficial effects of HIF-PHIs towards other disease states; for example, in ischemic hypoxic injury, infection/wound healing/inflammation, and inflammatory disease and atherogenesis [142,143].

Many reports require validation using well-characterized HIF-PHIs, although relatively strong evidence derives from reports showing protection with HIF-PHIs in colitis models [144,145,146].

This supports the mounting evidence for an anti-inflammatory role of HIF and HIF-PHIs [147]. For example, in mouse models of inflammatory colitis, animals with increased HIF-1α or PHD1 deletion are protected against different models of inflammatory colitis, while models with HIF-1α deletion are more susceptible [148,149,150].

“Clinical relevance remains speculative but new HIF-PHIs may provide benefits in a broad spectrum of disease states.”

## 5. Conclusions

Anemia is a common problem for the millions of people with CKD, and concurrent anemia is associated with an increased risk of morbidity and mortality. Treatment of anemia of CKD therefore has the potential to save many lives and improve QoL for many others. However, our current therapy has significant shortfalls in effectiveness. Treatment with iron helps in patients with concomitant iron deficiency, but iron overload may well lead to worse outcomes. Inflammation and lack of accessible iron stores in the face of total body iron overload, associated with very high ferritin levels, are major causes of ESA resistance, resulting in the need for increased doses of ESAs to achieve target hemoglobin levels, consequently with increased cost and potentially worsened outcomes as observed in the TREAT study. Moreover, the benefit of treating anemia with ESAs in patients with CKD has been called into question entirely, given that treatment of anemia in CKD with ESAs was shown to be inferior to placebo in the TREAT study. Against this background, patients with anemia of CKD are therefore currently treated simply to avoid symptoms and reduce risk of blood transfusion, but not to manage anemia and its associated risks.

Treatment with HIF-PHIs provides the potential for treating anemia of CKD with the possibility of gaining the benefits of reversing anemia while avoiding inherent dangers associated with high-dose iron and ESA therapy. Moreover, in reducing hepcidin levels, HIF-PH inhibition has the potential to increase access to existing iron stores and avoid the need to administer iron; potentially reducing risk of iron overload and bypassing the principal cause of ESA resistance and resistant anemia of CKD. Through sustained increases in EPO levels to physiologic, rather than supraphysiologic, levels, HIF-PHIs are anticipated to avoid the increased morbidity and mortality associated with the therapeutic use of ESAs.

Existing observational studies support the safety and benefit of increased HIF function; for example, lower cancer-related mortality is seen in populations living at relatively high altitude and, of these patients, those with ESRD have higher hemoglobin levels and better outcomes than those living at lower altitude.

A key barrier to providing full benefits with HIF-PH inhibition is the reported worse outcomes with the aggressive treatment of anemia of CKD with ESAs as evidenced in multiple studies. Based on this body of data, it is reasonable to conclude that normalization of hemoglobin is contraindicated. However, plausible hypotheses exist based on differences in the treatment effects of HIF-PHIs that suggest that HIF-PHIs could achieve normalization of anemia without the adverse outcomes as seen in recent studies of ESAs. Further clinical data are needed in this area.

HIF-PHIs may also have an important role in treating many disorders resulting from ischemia–reperfusion injury, including ischemic acute kidney injury, myocardial injury, and damage to transplanted organs. Furthermore, through effects on transient receptor potential channels, HIF-PHIs have the potential to improve outcomes in many other disease states.

As results from phase 3 trials become available to establish the efficacy and safety of HIF-PHIs in treating anemia of CKD, it will be possible to plan further trials readdressing, for example, if it is possible to achieve the benefits of normalizing hemoglobin in anemia without associated risks as demonstrated in trials of ESAs, and also whether the iron overload currently highly evident in the ESRD population can be avoided. Trials looking specifically at the effects of HIF-PHIs in patients with ESA resistance will be helpful. Finally, further trials could perhaps consider the use of HIF-PHIs in many other disease states where hypoxia plays a central role in pathophysiology. We firmly believe that through such interventions, HIF-PHIs will revolutionize the treatment of anemia in CKD as well as many other diseases. 

## Figures and Tables

**Table 1:** Impact of prolyl-hydroxylase domain (PHD), hypoxia-inducible factor (HIF), factor inhibiting HIF (FIH), and von Hippel-Lindau gene (VHL) mutations on cellular and tissue function.

Mouse Models		
**Gene**	**Outcome**	**Publication**
*PHD1*	Knockout (KO) protects from I/R injury in the liver and heart, reducing size of infarction and increasing scavenging of oxygen radicals, and protects against ischemic stroke	Schneider, M.; et al. *Gastroenterology* 2010 [28]
		Adluri, R.S.; et al. *Antioxid. Redox Signal* 2011 [29]
		Quaegebeur, A.; et al. *Cell Metab.* 2016 [30]
	Metabolic disturbance; KO promotes liver steatosis and insulin resistance, with increased glycolysis; attenuated hypercholesterolemia and hyperglycemia	Thomas, A.; et al. *Sci. Rep.* 2016 [31]
		Marsch, E.; et al. *Eur. Heart J.* 2016 [32]
	KO increases capillary and arteriolar density in response to ischemia	Rishi, M.T.; et al. *Microvasc. Res.* 2015 [33]
	KO increases hepatocyte proliferation and liver regeneration	Mollenhauer, M.; et al. *Langenbeck’s Arch. Surg*. 2012 [34]
*PHD2*	Conditional knockout (CKO) leads to increased angiogenesis and angiectasia	Takeda, K.; et al. *Circulation* 2007 [35]
	CKO increases EPO levels and erythropoiesis	Takeda, K.; et al.; *Blood* 2008 [36]
	CKO in EPO-producing cells leads to decreased bone density, while CKO in chondrocytes leads to increased bone density	Rauner, M.; et al. *J. Bone Miner. Res*. 2016 [37]
		Cheng, S.; et al. *Endocrinology* 2016 [38]
	PHD2 erythrocytosis	Arsenault, P.R.; et al. *J. Biol. Chem.* 2013 [39]
		Franke, K.; et al. *Blood* 2013 [40]
*PHD3*	KO leads to increased angiogenesis, with increased cardiac function and decreased fibrosis after ischemic injury	Oriowo, B.; et al. *Curr. Pharm. Des.* 2014 [41]
		Xie, L.; et al. *J. Mol. Cell. Cardiol.* 2015 [42]
	Regulation of neuronal apoptosis; dysregulation of sympathoadrenal development	Bishop, T.; et al. *Mol. Cell. Biol.* 2008 [43]
	KO leads to decreased neuronal apoptosis but decreased sympathoadrenal function	Taniguchi, C.M.; et al. *Nat. Med.* 2013 [44]
	Knockdown in glioblastoma cells and KO in astrocytoma cells; increased tumor growth	Henze, A.T.; et al. *Nat. Commun.* 2014 [45]
*FIH-1*	KO causes decreased weight, increased metabolic rate, resistance to hepatic steatosis, and high fat diet-induced weight gain (occurs also with KO in neuronal cells)	Zhang, N.; et al. *Cell Metab.* 2010 [46]
*HIF2A*	*HIF2A* erythrocytosis	Tan, Q.; et al. *J. Biol. Chem.* 2013 [47]
**Human Mutations**		
*PHD2*	Mutation causing decreased function causes congenital erythrocytosis	Percy, M.J.; et al. *Proc. Natl. Acad. Sci. USA* 2006 [48]
*HIF2A*	Mutation decreasing binding to PHD2 and VHL causes erythrocytosis	Van Wijk, R.; et al. *Haematologica* 2010 [49]
		Percy, M.J.; et al. *NEJM* 2008 [50]
*VHL*	Germline loss-of-function in VHL leading to erythrocytosis	Gordeuk; et al. *Blood* 2011 [51]

CKO: conditional knockout; I/R: ischemic/reperfusion; EPO: erythropoietin; KO: knockout.
